# Familial oral lichen planus in a 3-year-old boy: a case report with eight years of follow-up

**DOI:** 10.1186/s12903-020-01333-x

**Published:** 2020-11-26

**Authors:** Fang Wang, Ya-Qin Tan, Jing Zhang, Gang Zhou

**Affiliations:** 1 The State Key Laboratory Breeding Base of Basic Science of Stomatology (Hubei-MOST) and Key Laboratory of Oral Biomedicine Ministry of Education, School and Hospital of Stomatology, Wuhan University, Luoyu Road, Wuhan, 237 China; 2 Department of Oral Medicine, School and Hospital of Stomatology, Wuhan University, Luoyu Road, Wuhan, 237 China

**Keywords:** Case report, Family history, Oral lichen planus, Pediatric population, Rb-bFGF

## Abstract

**Background:**

Oral lichen planus (OLP) is a chronic mucocutaneous disease characterized by adult predominance and a prolonged course. However, it is rare in the pediatric population with familial aggregation.

**Case presentation:**

A 3-year-old boy presented with pain and irritation on the oral mucosa while contacting spicy food for 2 months. Oral examination showed widespread whitish reticular and papular lesions on the lips, the dorsum of the tongue, and bilateral buccal mucosa, with diffuse erosions covered with pseudomembrane on the buccal mucosa. The boy’s parents were examined to exhibit white reticular and plaque-like lesions on their oral mucosa. The three patients were clinically diagnosed as affected by OLP and histopathologically confirmed. The boy underwent topical treatment with recombinant bovine basic fibroblast growth factor (rb-bFGF) gel, and oral lesions gradually resolved and healed. Neither of his parents received treatment. During the subsequent follow-ups, none of three patients underwent any medical treatment. Fortunately, their lesions had almost faded over 8 years.

**Conclusions:**

Our case emphasizes that pediatric OLP should be recorded with family history. Besides, long-term periodic follow-up is recommended in pediatric patients with OLP for monitoring any changes in lesions.

## Background

Oral lichen planus (OLP) is a chronic T-cell-mediated mucocutaneous disease of unknown etiology [1]. The potential triggers, with a possible role in the pathogenesis, include autoimmunity, infective agents, microorganisms, vitamin deficiencies, psychological stress, and genetic predisposition [1, 2]. OLP is clinically characterized by well-defined and slightly raised white striae in the oral mucosa. Oral lesions are categorized as reticular, atrophic, erosive, papular, plaque, and bullous forms [1]. The typical distribution is almost symmetrical, frequently affecting the buccal mucosa, tongue, and gingiva. OLP’s histopathological features are epithelial hyperparakeratosis, basal cell liquefactive degeneration, and a band-like lymphocytic infiltrate in the lamina propria [1, 3]. Additional microscopic findings include atrophy, acanthosis, saw-tooth rete ridges, and colloid bodies. According to the World Health Organization (WHO), OLP is defined as an oral potentially malignant disorder (OPMD) [1]. A regular follow-up is essential to screen for changes that might indicate malignant transformation in OLP lesions [2].

OLP is uncommon in pediatric patients, and the etiology of pediatric OLP has not been well established [4]. Our previous study reported four pediatric cases in 674 patients with preexisting diagnosed OLP [5], while Alam and Hamburger documented six cases out of 1062 OLP patients [6]. Notably, pediatric OLP with familial aggregation is extremely rare. Since 2003, two pediatric OLP patients who exhibited a family history have been reported [7, 8]. Here, we present a case of a 3-year-old boy who was affected by OLP with familial aggregation, and his oral lesions disappeared almost spontaneously over 8 years.

## Case presentation

A 3-year-old boy was referred to the Department of Oral Medicine with a complaint of pain and irritation on the oral mucosa while contacting spicy food for 2 months. Relevant systemic diseases or medical histories were not available.

**Fig. 1:**
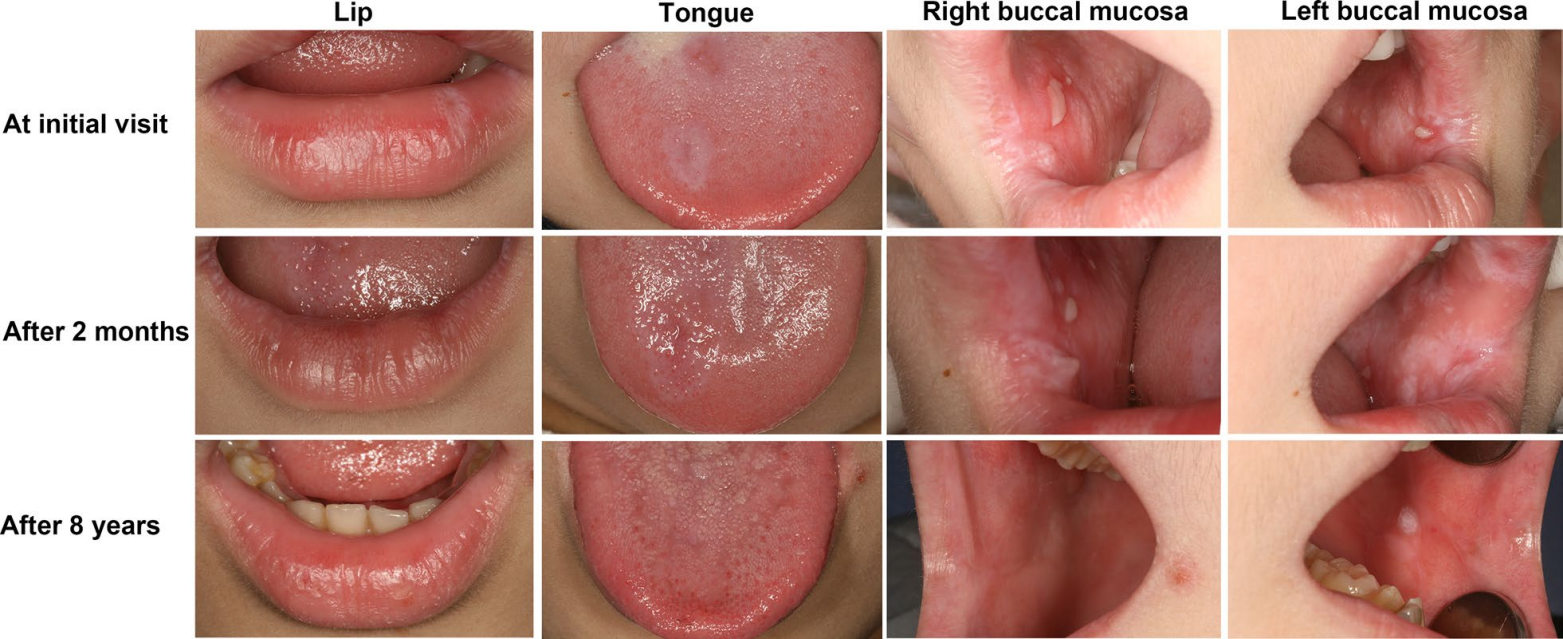
Clinical examination of the 3-year-old boy at the initial visit, and 2-month and 8-year follow-up visits

Whitish reticular and papular lesions were widespread on the lips, the dorsum of the tongue, and bilateral buccal mucosa on the oral examination, respectively (Fig. 1). The lesions were non-scrapable, non-indurated, and non-tender. There were diffuse pseudomembranous erosions on the buccal mucosa. Irregular hyperpigmented areas were observed on the back and the right arm on the extraoral examination. There were no lesions on the nails. An incisional biopsy from white lesions on the right buccal mucosa revealed findings consistent with OLP.

**Fig. 2:**
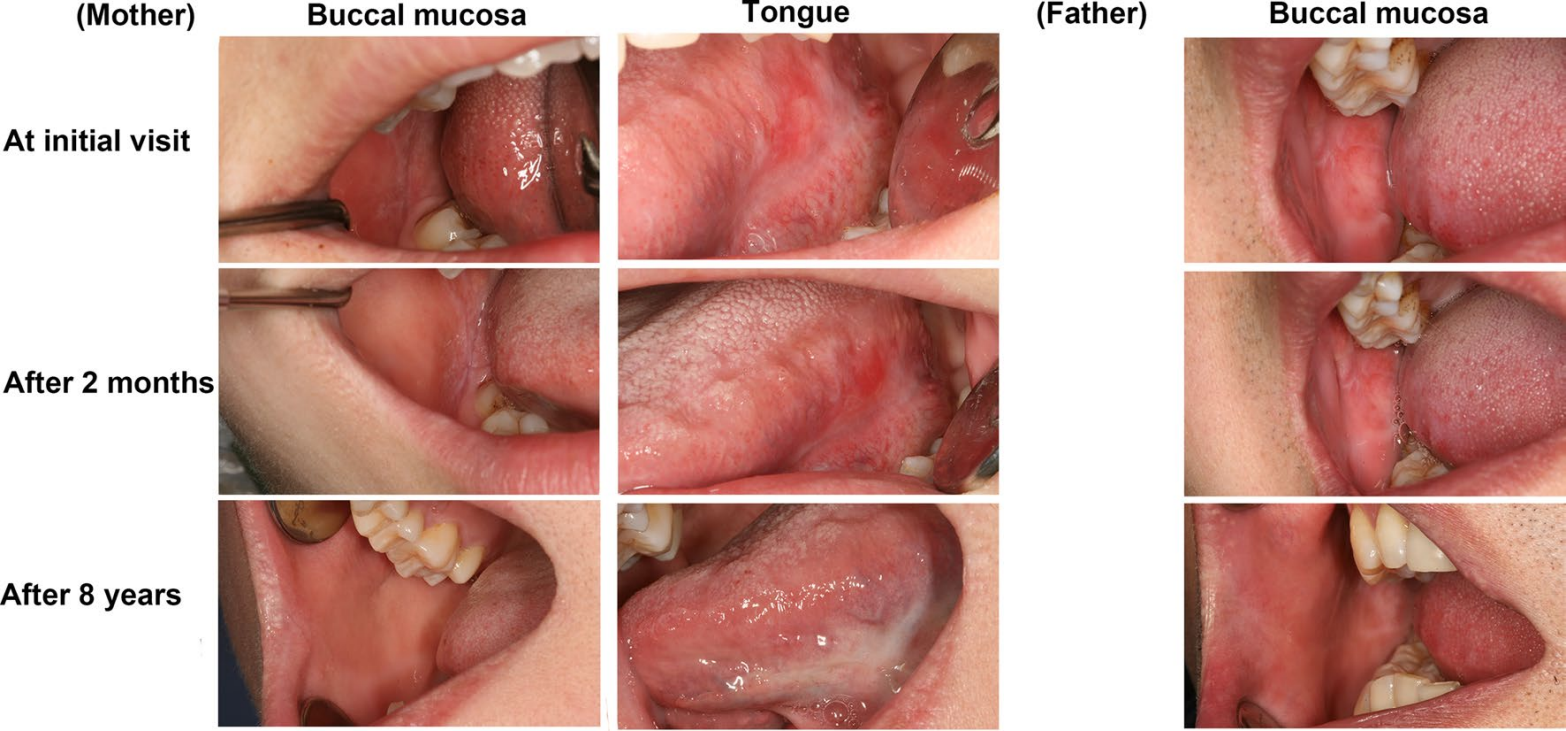
Clinical examination of the patient’s parents at the initial visit, and 2-month and 8-year follow-up visits

**Fig. 3:**
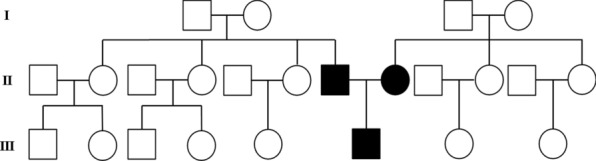
The pedigree of the family

Interestingly, the boy’s mother (29 years old) exhibited white reticular and plaque-like lesions on the right buccal mucosa and atrophy on the left ventral surface of the tongue (Fig. 2). His father (34 years old) had similar white lesions on the right buccal mucosa (Fig. 2). Incisional biopsy from the right buccal mucosa of them confirmed the diagnosis of OLP. No other relatives of this family had OLP lesions, and this information was collected from parents’ narration (Fig. 3). Relevant medical history and systemic diseases were not available.

Based on the principles of the Food Frequency Questionnaire (FFQ) [2, 4] and Chronic Oral Mucosal Diseases Questionnaire (COMDQ) [5], we designed a questionnaire concerning the family lifestyles, including dietary habits, prolonged exposure to poor dentary conditions, and mental stress. The questionnaire results showed that the family members preferred food with a strong taste, including spicy, smoked, and fried dishes.

The boy underwent topical treatment with recombinant bovine basic fibroblast growth factor (rb-bFGF) gel for 2 months, and the symptoms and signs gradually resolved. Then, the rb-bFGF gel was applied for another week, and the erosions healed. Neither of his parents took treatment. The three patients were recommended regular annual follow-ups in the long-term care for monitoring any changes in oral lesions. During the subsequent follow-ups, the oral mucosa of them clinically manifested asymptomatic whitish lesions; they did not undergo any medical treatment. Fortunately, their lesions almost faded over 8 years. Only slightly white plaque-like lesions were found on boy’s left buccal mucosa and dorsum of the tongue.

## Discussion and conclusions

OLP is a rare phenomenon in pediatric patients, and only a few cases have been reported in the literature to date [4]. The prevalence of pediatric OLP varies from 0.03 to 0.56%, compared with approximately 2.2% of the general population [1, 5, 6, 9]. Cascone et al. reviewed 26 pediatric OLP cases between 1966 and 2015 and described that the average onset age was 10.41 ± 3.24 years [4]. The case reported here was diagnosed with OLP at only 3 years of age, the youngest case documented to date.

OLP manifests variability in clinical presentation, ranging from a classic lace-like network of slightly raised white lines with multifocal symmetrical distribution to erythematous and painful erosive lesions. Many studies have suggested that oral lesions of pediatric patients have the classic clinical presentation of OLP [4, 10, 11]. Similar to adults, asymptomatic reticulation is the dominant form of pediatric OLP [4]. Besides, the buccal mucosa is the most common site affected by OLP in pediatric patients [11]. In our case, the pediatric patient presented multifocal, symmetrical reticular and papular lesions accompanied by scattered erosions, suggesting a provisional diagnosis of OLP, which was confirmed by histopathological findings.

Although lichen planus is usually a sporadic disease, a familial form has been reported in 1 to 4.3% of pediatric patients. Studies have shown that the most notable features of familial OLP include early age of onset, widespread presentation on the oral mucosa, frequent clinical relapses, and increased disease severity [7, 12, 13]. Increasing evidence has found linkage of familial series with human leukocyte antigen (HLA) genes, such as HLA-B7, HLA-BR10, and HLA-D [8, 12, 14]. Currently, only two case reports are available on OLP’s inheritance in first-degree relatives among the pediatric population. One described a 12-year-old girl with whitish streaks on the oral mucosa, whose mother also exhibited whitish streaks on the buccal mucosa and violaceous patches on the tongue [7]. The other reported that an 11-year-old boy was affected by an erythematous lesion with a violaceous hue on the right half of the tongue, his father and grandmother both had oral involvement confirmed as OLP [8]. However, in our case, the condition was diagnosed at 3 years of age, and the clinical presentation was more complex and varied. The patient’s oral lesions affected the lips, the tongue, and the buccal mucosa, which were multifocal and have rarely been reported. Additionally, the boy’s parents were clinically and histopathologically diagnosed as affected by OLP, which has not been reported in the familial form before.

According to an updated review about OLP treatment, the aims of therapeutic management are eliminating painful symptoms, promoting healing of erosive lesions, reducing the risk of malignant transformation, and prolongating symptom free periods [15]. For erosive OLP, corticosteroids were recommended as first-line treatment in consensus guidelines [15, 16]. However, systemic or topical application of corticosteroids present various adverse effects, containing oral candidiasis, a smarting sensation in the mouth, gingival tenderness, insomnia, mood swings, fatigue, and water retention [16–18]. Moreover, there is no definitive protocol of corticosteroid therapy for children aged three. Here, we tried to treat the child with safer drugs to avoid corticosteroids’ adverse effects. bFGF is a pleiotropic cytokine that promotes angiogenesis and neurotrophy, and is important in the maintenance of mucosal integrity [19]. Pharmacological studies have shown that rb-bFGF stimulates mRNA, DNA, and protein synthesis in fibroblasts and endothelial cells [20]. Besides, rb-bFGF promotes keratinocyte division, epidermal regeneration, and granulation tissue formation, thus repairing tissue damage and accelerating wound healing [20]. Moreover, in clinical practice, we have used rb-bFGF gel to treat ulcerative and erosive lesions effectively. Li et al. also reported that topical application of rb-bFGF had positive effects over ulceration healing for a six-year old patient [21]. Therefore, in this case, the boy underwent rb-bFGF gel instead of corticosteroids. Fortunately, the patient’s oral erosions healed gradually, without adverse effects.

OLP is one of OPMDs according to the WHO criteria, with a malignancy potential rate of 0.4 to 12.5% [1]. Our previous study reported four cases with malignant transformation, which occurred at sites previously diagnosed by clinical examination as erosive or atrophic OLP [5]. Thus, continued clinical follow-ups are essential for monitoring OLP’s malignant transformation, especially when there are erosive and atrophic lesions. Alrashdan et al. recommended that regular follow-up ranging from every 2 months to once a year might be accepted in the long-term care for OLP patients [2]. Malignancy risk in children has rarely been reported, however, it is still necessary to consider the possibility of malignant transformation in pediatric OLP. Therefore, we suggest that pediatric populations in OLP also need regular annual follow-ups. In this case, the boy and his parents had good prognosis and their lesions had almost faded over 8 years.

In summary, we reported a case of a 3-year-old boy affected by OLP and with familial aggregation, whose lesions faded spontaneously during an 8-year follow-up. OLP lesions are recalcitrant and pre-malignant, particularly when erosive or atrophic lesions are present. Therefore, regular annual follow-ups are recommended in pediatric patients with OLP to monitor any changes in oral lesions.

## Data Availability

The datasets used and/or analyzed during the current study are available from the corresponding author on reasonable request.

## Abbreviations

COMDQ: Chronic Oral Mucosal Diseases Questionnaire
FFQ: Food Frequency Questionnaire
HLA: Human leukocyte antigen
OLP: Oral lichen planus
OPMD: Oral potentially malignant disorder
rb-bFGF gel: Recombinant bovine basic fibroblast growth factor gel
